# Œsophageal Obstruction.—IV

**Published:** 1908-08-29

**Authors:** 


### Surgery.

### OESOPHAGEAL OBSTRUCTION.—IV.

### THE OPERATION OF GASTROSTOMY.

When it has been finally decided that the patient's condition calls for gastrostomy, and that the proper moment for performing it has arrived, the operation should be done without delay. Whatever method be chosen, there is one question which nearly always arises, and that is, will the patient stand a general anæsthetic? The danger is more often apparent than real, and the question is prompted by the emaciated aspect of the patient, which is the outcome of the fact that he has not been obtaining sufficient nutrition for some time past. As a matter of fact, experience teaches us that debilitated patients take an anæsthetic as well as, if not better than, more robust ones. But if, in the opinion of a skilled anæsthetist, there is the least risk involved, then it is better to perform the operation under local anæsthesia. A mixture of 4 per cent. eucaine, and a solution of adrenalin, one in a thousand, will be found most satisfactory. Three or four minims of this are injected at several points of the circumference of the area of operation. Half a drachm may be given in this way, a few minutes before the commencement of the operation; the anæsthesia so produced may be tested from time to time with the point of the scalpel, and as soon as sensation is abolished, the incision may be made.

The operation which will be found most generally satisfactory is that known as Frank's or Albert's method. A vertical incision is made through the left rectus abdominis muscle, one inch to the left of the middle line, and about three and a half inches long. Through this wound a cone of stomach is pulled out. A second incision is then made just over, and parallel to, the costal arch on the left side. This is carried down as far as the anterior layer of the sheath of the rectus muscle, and a communication is made between the two by burrowing between the fibres of the rectus muscle. A stout silk suture is then put through the apex of the cone of exposed stomach wall. Now in most text-books of surgery one is advised to pass this guiding suture through the sero-muscular coat of the stomach only, on the supposition that if the needle passes through the cavity of the stomach the contents will leak through and peritonitis will supervene. As a matter of fact this does not occur; and moreover, there is an excellent reason for passing the suture through all the coats. If this is not done, and traction is put upon the guiding suture, the mucous coat is not drawn through with the rest, and great trouble may be experienced subsequently in opening the stomach; and it may actually happen that the catheter is inserted between the mucous and the sero-muscular coats with disastrous results. The guiding suture having therefore been passed through all coats of the stomach, it is drawn through the tunnel in the rectus muscle until about half an inch of the cone protrudes through the smaller opening. The right edge of the base of the cone is then fixed in position by suturing it to the cut edge of the sheath of the rectus. It is well not to include the peritoneum in this line of sutures. The first incision is then closed, and the apex of the cone is sutured to the edges of the second incision by means of four fine silk sutures. Here again the writer ventures to diverge from the accepted descriptions of the operation. One is usually advised to defer making the incision into the stomach for twenty-four or forty-eight hours in case gastric contents escape and infect the surrounding peritoneum. But if the operation has been properly done, there is not the least likelihood of this occurring, and it is far easier to make certain that the stomach has really been opened at the time of the operation than it is forty-eight hours later, when the condition of the structures is much modified, even when the case has pursued a completely aseptic course. The writer remembers a case which was under his care as house-surgeon. A gastrostomy was performed, and what was taken to be the stomach was opened forty-eight hours later, and food was introduced by means of a catheter. The patient developed symptoms of general peritonitis, and died. It was found at the autopsy that the catheter had passed between the stomach wall and the edge of the wound, and the food had been introduced into the peritoneal cavity. Perhaps the writer is prejudiced by this experience, but it seems to him that the risk of not entering the stomach is greater than that of infecting the peritoneum at the time of operation. The more one sees of intestinal surgery, the more one must be convinced that the excessive care taken to avoid puncturing the mucous coat of the intestine with a needle is somewhat unnecessary, if one can judge by the successful results of such operations as gastro-jejunostomy, in which the needle has certainly pierced all coats of the intestine during the introduction of the posterior row of stitches.

When the stomach has been opened, whether this be done at the time of operation or later, a soft No. 12 catheter is introduced into it, and left *in situ* for three or four days. After this time firm adhesions will have formed, and the catheter need only be inserted for the purpose of feeding. Thus food can be introduced into the stomach, and the patient is prevented from dying of starvation and, in some cases, is enabled to live in comparative comfort until he dies from exhaustion or cachexia.

The operation described above is the one in common use, and is possible in nearly every case. Sometimes, however, the stomach is either very contracted or fixed by extension of the original disease. In either case a cone of stomach wall cannot be pulled out in the manner described. In these cases the operation known as Witzel's has distinct advantages. Briefly it consists in making a tube of stomach wall round the end of a catheter, whose apex is inserted into the stomach at the operation. It is, however, more difficult than Frank's.

